# Japanese Encephalitis: A Brief Review on Indian Perspectives

**DOI:** 10.2174/1874357901812010121

**Published:** 2018-08-31

**Authors:** Reshma Kulkarni, Gajanan N. Sapkal, Himanshu Kaushal, Devendra T. Mourya

**Affiliations:** ICMR-National Institute of Virology, 20-A, Dr. Ambedkar Road, Pune-411001, India

**Keywords:** Japanese encephalitis virus, Notifiable disease, Vector-borne disease, Diagnosis, Prophylactics, India

## Abstract

**Introduction:**

Japanese encephalitis (JE) is recently declared as a notifiable disease in India due to its expanding geographical distribution. The disease notification facilitates effective implementation of preventive measures and case management.

**Expalantion:**

JE is a vector-borne disease that can be prevented by vaccine administration. It is caused by Japanese encephalitis virus (JEV), belonging to family *Flaviviridae*. Amongst the known etiological viral encephalitis agents, it is one of the leading viral agents of acute encephalitis syndrome in many Asian countries where it is identified to cause substantial morbidity and mortality as well as disability. Globally, it is responsible for approximately 68,000 clinical cases every year.

**Conclusion:**

In the absence of antivirals, patients are given supportive treatment to relieve and stabilize. Amongst available control strategies; vector control is resource intensive while animal and human vaccination are the most effective tool against the disease. This review highlights recent progress focusing challenges with diagnosis and prophylactic interventions.

## 1 INTRODUCTION

Japanese encephalitis virus (JEV) is an arthropod-borne flavivirus, which has a wide distribution in many countries of Asia, Western Pacific and in northern Australia. Twenty-four countries from South-East Asia, Western Pacific regions are endemic for this disease and more than three billion people are at risks of infection. Symptomatic cases are uncommon and occur in approximately 1 in 250 subclinical infections. Japanese encephalitis (JE) is a major threat with case fatality rate up to 30% among those with disease symptoms. The infection causes a spectrum of clinical illness that begins with flu-like symptoms, neck stiffness, disorientation, coma, seizures, spastic paralysis and eventually death. JEV is one of the major public health problems not only because of a large number of deaths but also due to severe neuro-psychiatric sequelae that necessitates lifelong support amounting towards considerable socioeconomic burden [1, 2]. An outbreak in India (2005) resulted in 1700 reported deaths mostly among children, accentuated the sustained burden of disease in developing countries [3, 4]. The effectiveness of vector control strategies is limited due to the complex eco-epidemiology of the virus. Vaccination is the most effectual means of prevention, where JEV is a major public health problem. 

This review provides an update on diagnostics methods, prophylactic as well as therapeutic options for the control and the progress that has been made in vaccine development. 

### 2 TRANSMISSION

The disease principally occurs in rural agricultural areas where vector mosquitoes breed in the close proximity with pigs, wading birds and ducks. The natural transmission cycle involves multiple mosquito species from genus *Culex* whereas pigs, birds and bats are the susceptible reservoir hosts. Humans as well as equines are considered as dead-end host since the viremia in peripheral blood is low and transient. The most important vector *Culex tritaeniorhynchus* is associated with agricultural practices like rice cultivation or irrigated crop fields [5]. The increase in JEV activity in newer areas has been attributed to the increase in human population, rice fields and pig farming [6]. Moreover, ardeid birds are considered responsible for the long-distance propagation of JEV and act as a reservoir for the disease [7].

Domestic pigs serve as key virus-amplifying host as they develop high viral as well as and long-lasting viremia after natural infection with JEV and facilitate transmission to humans living in their close proximity [8]. Horses and other non-avian vertebrates are the incidental dead-end hosts as they do not develop sufficient level of viremia to infect new mosquitoes [9]. Transmission is principally associated with the rainy season in Southeast Asia, however, can happen throughout the year in tropical regions. In the temperate regions of China, Japan, the Korean peninsula and eastern parts of the Russian Federation, transmission occurs primarily during the summer and autumn. Two vital determinants of vector density *viz.* precipitation and temperature influence the disease burden of JEV [10].

### 3 THE PATHOGEN

The size of JEV is approximately 50 nm spherical particles containing electron-dense core (approximately 30 nm) enclosed by a lipid envelope. The genome is single-stranded positive-sense RNA of approximately 11 kb length. The virus consists of a lipoproteinous envelope surrounding the nucleocapsid and core; the genome is packaged in the capsid which in turn is coded by a capsid protein (C). The outer membrane of JEV comprises an envelope protein (E) that facilitates the virus entry into host cell. E protein is also recognized as a protective antigen. Additionally, the genome encodes for a membrane protein (M), and seven non-structural proteins (NS1, NS2A, NS2B, NS3, NS4A, NS4B, NS5) of which NS3 is a helicase while NS5 is a polymerase [11, 12].

While protective immunity is conferred by passive transfer of antibodies against NS1, the protein can direct complement-mediated lysis of infected cells *via* interaction with the cell-surface-associated form of NS1. NS2A is perhaps associated in the coordination of the shift between RNA packaging and RNA replication. The NS2B protein is a small, membrane-associated protein. The exact function of NS4A and NS4B is still unknown.

### 4 GENOTYPES

**Fig. (1):**
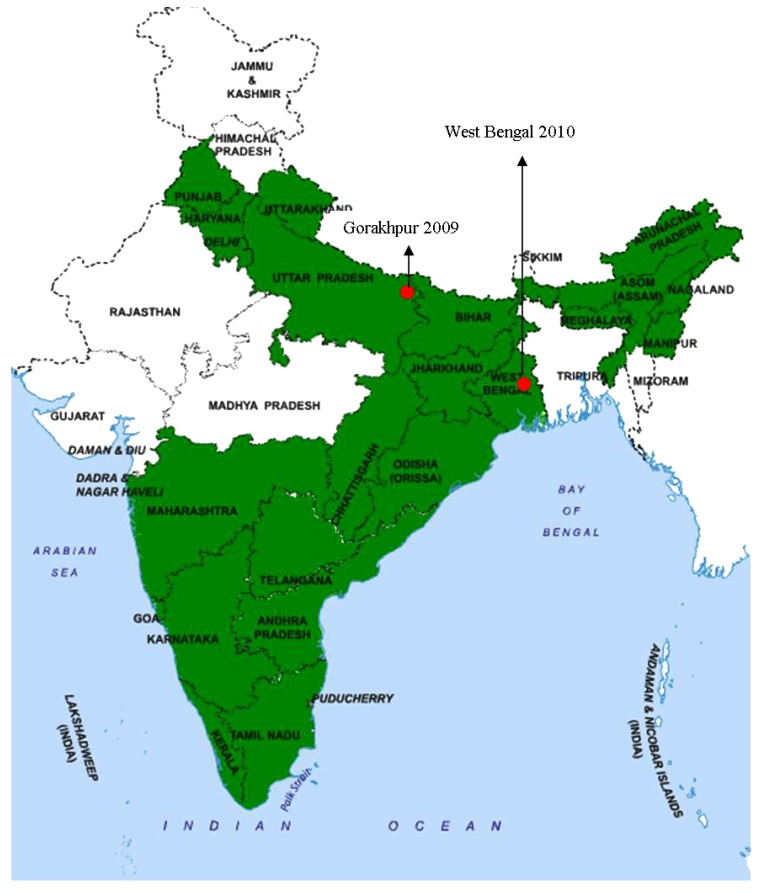
** Distribution of Japanese encephalitis in India.**  States affected by Japanese encephalitis  Recent introduction of Genotype I in India

The nucleotide sequencing studies on the partial or complete genome of JEV revealed that it has five JEV genotypes, G-I to G-V [13]. The G-I and G-III genotypes are present mostly in temperate epidemic areas, whereas G-II and G-IV are reported in tropical endemic regions [14]. JE is endemo-epidemic in several countries with reports of G-I replacing G-III as the dominant genotype in numerous regions including India (Fig. **1**) [15-17]. All genotypes of JEV form a single serotype [18]. G-I includes isolates from northern Thailand, Korea, Cambodia while G-II includes isolates from southern Thailand, Indonesia, Malaysia and Northern Australia. G-III encompasses isolates mostly from temperate regions of Asia (China, Taiwan, Japan and the Philippines). G-IV consists of virus isolates chiefly from Indonesia. In 1952, another distinct strain of JEV was isolated from Muar, Malaysia (Muar strain) that has been considered as fifth genotype (G-V), based on phylogenetic analysis and cross-neutralization. Indonesia-Malaysia region is the sole geographic area where all genotypes are found [19].

### 5 EPIDEMIOLOGICAL PATTERN AND DISEASE INCIDENCE IN INDIA

Two epidemiological patterns are documented for the disease. JE outbreaks coincide with monsoons and post-monsoon period due to a marked increase in vector density. However, in endemic areas, sporadic cases may occur throughout the year. Karnataka and Andhra Pradesh experiences two epidemics every year, first from April to July that is quite severe while second from September to December being milder similar to the rest of India [20].

In India, JE is a leading pediatric health issue and epidemics have been reported from many regions since 1955. The earliest evidence of JEV in India was obtained through the studies conducted in 1952 [21]. A major outbreak occurred in the Bankura district of the state of West Bengal in 1973 [22]. Since then, the virus was found active almost in every part of India and outbreaks have been reported regularly. The most affected states comprise of Andhra Pradesh, Assam, Bihar, Haryana, Karnataka, Kerala, Maharashtra, Manipur, Tamil Nadu, Orissa, Uttar Pradesh and West Bengal. Epidemics are reported from union territories of Goa and Pondicherry as well [23].

The state of Uttar Pradesh (UP) has been under constant surveillance for JEV activity since 1978 [24]. The longest epidemic of viral encephalitis was reported from Gorakhpur district, UP between July and November 2005 [25]. A total of 5,737 cases from 7 districts of eastern UP were reported of which 1,344 persons succumbed to the disease. The regions of eastern UP (Gorakhpur and Basti divisions) are conducive for the spread of the virus due to the abundance of paddy fields, a bowl-shaped terrain and are also prone to annual flooding. An outbreak of encephalitis in Lakhimpur Khiri district was investigated for the identification and characterization of the etiologic agent that showed homology with JEV strain GP78 [26]. In 1990, the epidemic among humans was first reported in Haryana [27]. Later, human cases were reported regularly in this state [28-30]. JE has been reported in newer areas, signifying the spread of virus owing to altered land use patterns as well as vector adaptation. In the absence of rural travel, JEV activity was reported from New Delhi [31].

The virus activity was reported regularly from states of northern and northeastern parts of India as well as its spread to naive non-endemic regions of the country [32]. Recently, India witnessed another large outbreak in Malkangiri during 2012 and Manipur in July 2016 [33, 34].

An unexplained acute neurologic illness affecting children with high case-fatality rates was reported from Muzaffarpur district of Bihar since 1995. The cause of illness was attributed to infectious encephalitis, extreme heat and humidity causing heat stroke as well as exposure to pesticides [35, 36]. A hypothesis linking this disease with the cultivation of litchi fruits was also proposed [37]. However, the disease was ascribed to the presence of hypoglycin A or methylenecyclopropylglycine (MCPG) – present in litchi that can cause hypoglycemia and metabolic derangement [38].

### 6 DIAGNOSIS

Clinically, it is difficult to distinguish JE from other cases of encephalitis; cases of acute encephalitis syndrome (AES), therefore laboratory confirmation is necessary in such circumstances. In JE cases, viremia is transient and infection is asymptomatic. Several assays have been developed for detection of antibodies induced by natural infection or vaccination [39]. Serological tests comprise of plaque reduction neutralization test (PRNT), the hemagglutination inhibition (HI) test, an indirect immunofluorescence assay (IFA) and an enzyme-linked immunosorbent assay (ELISA). A multitude of tests based on nucleic acid detection have been explored for JEV detection in humans as well as swine population [40-42]. However, for laboratory-based surveillance following markers are used for confirmation of the disease.

1. The ideal method for laboratory confirmation is testing cerebrospinal fluid (CSF) or serum for JEV-specific IgM antibody.
2. Plaque reduction neutralization test.
3. Virus isolation.
4. Nucleic acid amplification.

### 7 ENZYME-LINKED IMMUNOSORBENT ASSAY (ELISA)

The JEV-specific IgM antibody capture ELISA (MAC-ELISA) has now become the first-line diagnostic assay recommended by WHO for detection of acute infections [43]. It is a simple platform that is more suitable for testing large numbers of specimens rapidly and is considered as a corner stone in clinical settings. ICMR-National Institute of Virology (NIV), Pune, India has developed a highly reliable IgM capture ELISA kit for rapid diagnosis of JEV in serum or CSF specimens. Additionally, this kit can efficiently capture IgM against both genotypes i.e, GI and GIII (ICMR-NIV, Pune unpublished data). A number of other ELISA kits are commercially available like Panbio JE-Dengue IgM Combo ELISA (Inverness Medical, Brisbane, Australia), InBios JE Detect (InBios International, Inc. Seattle, USA); and the JEV Chex kit (XCyton LLC, Bangalore, India).

### 8 PLAQUE REDUCTION NEUTRALIZATION TEST (PRNT)

PRNT is considered as a gold standard in flavivirus diagnosis. To discriminate between potentially cross-reactive antibodies with other flaviviruses, PRNT is the test of choice [44]. A fourfold increase in IgG titre in acute and convalescent sera is considered as a confirmatory test. This rise in antibody titre rules out the possibility of previous exposure to the virus.

### 9 VIRUS ISOLATION

Lack of animal models is a long-standing problem for virus isolation. Isolation can be done in mice using intra cerebral route. However, transient and low-level viremia is observed in JE infection, therefore, the isolation of virus is not a method of choice for diagnosis in clinical specimens.

### 10 NUCLEIC ACID AMPLIFICATION

The RT-PCR tests, quantitative PCR (TaqMan), restriction fragment length polymorphism (RFLP) analysis are useful molecular assay tests as they are very specific, sensitive and can detect low viral copies in acute or early phase of infection [45]. An RT-LAMP-LFD assay that combines reverse transcription loop-mediated isothermal amplification (RT-LAMP) with a lateral flow dipstick (LFD), is of great importance in diagnosis of JEV infection as it is a fast, highly sensitive and specific assay [46].

### 11 CONTROL AND PREVENTION STRATEGIES

JE is a leading public health problem in India due to its complex eco-epidemiology. The JE cases thus represent the tip of the iceberg compared to a large number of subclinical infections. Thus, the incidence of JEV cases is not the actual indication of the at-risk population. Considering the gravity of the problem of AES & JE in the country, Government of India has formulated a multipronged strategy to reduce the disease burden as well as to prevent mortality, morbidity and disability. The strategy includes JE vaccination in affected districts and strengthening of surveillance programs. Additionally, vector control, case management, timely referral of serious and complicated cases are also done. Appropriate sanitation facilities, as well as access to safe drinking water, are also aimed at this strategy. Provision for physical, medical, neurological and social rehabilitation is included to estimate disability burden due to JE. Moreover, improvement in the nutritional status of children at risk has also been designed [25].

Mosquito control has not been found effective *per se.* There is also a mammoth need of newer eco-friendly pesticides for the effective control of vectors. Immunization of pigs as well as avoiding human exposure to the infected mosquitoes seems feasible but is a short-term solution in high-risk endemic zones. Therefore, vaccination is the only effective strategy to control JE infection. The above-mentioned guidelines facilitate our preparedness for the risks associated with JEV. Thus control of JEV is a feasible goal and can reduce the disease incidence.

### 12 VACCINES

Vaccination is the most cost-effective therapeutic intervention. Elimination of the virus is not possible, since, it is maintained in an enzootic cycle involving mammals and birds. Therefore immunization is most effective for prevention and achieving long-term protection.

Advancements in the availability and development of JEV vaccines have rejuvenated the scenario for JE control. The four major types of safe and effective JE vaccines are:

### 12.1 Inactivated Mouse Brain-Derived Vaccines

JE-VAX is a mouse-brain derived inactivated virus vaccine manufactured in Japan (1930s) that was internationally distributed by Sanofi-Pasteur in the US and Europe [47]. Purified mouse brain-derived wild-type Nakayama or Beijing-1 strains were used for vaccine preparation. Three doses were recommended for travellers while for the children (1-3 years) 2-doses were administered in endemic regions. Long-term protection was observed in children while in the travellers a reduced amount of protection was observed (<1 year for 70% of vaccinees). Facial as well as oropharyngeal angioedema, urticaria contributed towards the adverse reactions post-vaccination in the travellers from European and North American region [48]. Fatal cases of acute disseminated encephalomyelitis were reported infrequently in children residing in the endemic locales and in travellers [49]. In 2005, the use of this vaccine was stopped and the vaccine stock was ceased due to increase in cases of acute disseminated encephalomyelitis associated with the vaccine.

### 12.2 Inactivated Vero Cell Vaccines

P3 strain of JEV grown in Primary Hamster Kidney cells was used for the preparation of inactivated JE vaccine. This vaccine was solely manufactured in China and was the principal JE vaccine till 2000. At peak production, approximately 70 million doses were distributed annually. This has decreased since the late 1990s to about 13 million in 2004 due to the availability and accessibility of live attenuated SA 14-14-2 vaccine [50].

### 12.3 Live Attenuated Vaccines

SA 14-14-2 strain propagated in Vero cells as well as in primary hamster kidney (PHK) cells were used for the manufacture this vaccine in China [47] in order to get wider licensure and compliance with the WHO production standards. An enhanced screening for the presence of adventitious agents in the vaccine was done. This vaccine was recently licensed in South Korea, Nepal, Sri Lanka and India. It is immunogenic, elicits broad protective immunity and is effective as well as safe for children. The vaccine efficacy is about 100% after two doses when administered at one or two years of age.

### 12.4 Chimeric Vaccines

Licensed in China, Thailand and India, ChimeriVax™-JE is a single dose lyophilized formulation of a recombinant, attenuated, chimeric virus that consists of structural genes (Pre-membrane and E) from SA 14-14-2 strain. These structural genes were incorporated into the backbone of attenuated strain of yellow fever (YF) virus YF 17D. It is effective in the pediatric population in endemic zones and can be in integrated into the national immunization program [51]. The new collection of safer and effective JE vaccines like, SA 14-14-2, IXIARO^®^, and IMOJEV™ can considerably decrease the burden of the disease. Since the availability of these vaccines for the population at-risk remains inadequate, there was an urgent requirement for the development of newer vaccines against JEV infection.

### 12.5 Vaccination in India

Vaccination remains the most effective preventive strategy for JEV control due to its complex eco-epidemiology. Mass vaccination with SA 14-14-2 was started in phased manner subsequent to the major outbreak in 2005. The vaccine was administered to children between the age group of 1 to 15 years. 132 districts have already been brought under JE vaccination as part of Universal Immunization Programme (UIP) [25]. In 2013, an additional dose of SA 14-14-2 vaccine at 9 months of age along with measles vaccine was added in UIP. So far, 155 out of 181 identified JE endemic districts are covered under JE campaign and more than 10 crore children were immunized in the course of vaccination campaigns.

Until 2013, the country was importing the vaccines from China. Considering the problems in the sustained vaccine supply and funding, several options have been offered for consideration. Vaccines are being developed by Shanta Biotechnic Pvt. Ltd., Hyderabad, Biological E Ltd., Hyderabad and Indian Immunologicals Ltd., Hyderabad as well as Bharat Biotech Hyderabad [52].

The JENVAC is an inactivated Vero cell-derived vaccine prepared from an Indian strain of the JEV. It is the first indigenously developed vaccine that is safe and highly effective against all known strains of JEV. The seroconversion rates were more than 90%. This vaccine can elicit protective responses with either a single or two doses is an added advantage for JE endemic countries. The vaccine has been found safe and efficacious for the age group between 1-50 years in Indian population [53].

### 13 ANTIVIRALS

Despite the availability of a few licensed vaccines, approximately 68,000 cases of JE occur annually across the globe [2]. Due to lack of approved antiviral prophylaxis for the infection mitigation; challenges remain for the prevention of mortality and morbidity. This situation necessitates the development of safe and cost-effective antivirals. Long and arduous efforts were made in the pursuit of suitable antivirals against JE. However, there is no FDA approved antiviral available for human use till date. Therefore, JE cases are managed by supportive care.

Several anti-JE drug trials have been undertaken [54]. The therapeutic efficacy of the antioxidants like minocycline, arctigenin, fenofibrate and curcumin are reported earlier [55]. Clinical trials with dexamethasone [56], IFN-α 2a [57], and ribavirin [58] did not show encouraging results. However, N-methylisatin-beta-thiosemicarbazone derivative (SCH 16) has been demonstrated to be effective in inhibiting JEV *in vitro* [59]. In another study, minocycline, a semi-synthetic tetracycline reduced the degree of neuronal damage seen in JEV infection in neuronal cell culture, partly by inhibiting oxidative stress [60]. Furthermore, minocycline demonstrated to maintain the integrity of blood-brain barrier following JEV infection [61]. A clinical trial with minocycline carried out in the state of Uttar Pradesh, India was found to be effective in curtailing the clinical symptoms such as fever, convulsions and duration of hospitalization [58]. A recent report underscores the utility of Bispidine-amino acid conjugates for its inherent scaffolding property in treating JEV infection [62]. Furthermore, in the absence of anti-JE drug, the strategy of combination therapy could be an interesting approach. An effective, safe and readily available antiviral drug is the need of the hour to control the mortality rate in the endemic areas across the world.

## CONCLUSION AND PERSPECTIVES

JE poses a menace to areas beyond its conventional geographical boundaries. Due to international travel, potential dispersal of the mosquitoes, climate change and urbanization, the virus is spreading into immunologically naïve populations. Control of JEV is thus challenging due to its complex eco-epidemiology. One hundred and seventy-one districts from nineteen states have been reported for JE and acute encephalitis syndrome (AES) in India [63]. With a view to address this issue in the country, Ministry of Health & Family Welfare has made disease notification mandatory for public health facilities since 21^st^ September 2016 [64]. Notification of the disease is an important source of information for the surveillance systems intended for the disease prevention and further spread. Furthermore, it provides a direction for the collection of accurate and useful disease burden data. The disease burden is not only associated with the acute disease, but also with the devastating after effects such as sequelae and long-term disability.

Accessibility to efficient, safer vaccines for pediatric use is critical as utility and availability of the mouse-brain-derived vaccines has noticeably decreased. Second generation tissue culture based vaccines with smaller and easier schedules are available globally [4]. The live-attenuated SA 14-14-2 JE vaccine has been added in national immunization programs recently. The associated problem in immunization program is the expense, logistics and sustained supply wherein they are required most. Development of novel, safe and affordable JE vaccines is imperative to expedite the pursuit for an effective modality with higher safety levels, efficacy, shorter dosing schedules that will meet the needs of changing genotypes [65]. A recent addition of JENVAC; inactivated cell culture-based, vaccine developed from Indian strain has established to be non-inferior to other commercially available vaccines. This vaccine may be an additional armor to reduce the incidence of JEV in India.

Moreover, due to lack of efficient antiviral treatment, diagnosis and prevention are the highest priorities that would greatly reduce the disease burden. Ecological control is unrealistic due to vector abundance and expense. Besides, it may have unanticipated harmful ecological effects. Additionally, systematic surveillance work on vector species, density as well as seroprevalence in pigs needs to be actively pursued in support of detection and providing alert for predicting outbreak in areas. Due to the recognition of JE as a notifiable disease, commitment for its prevention and control as well as the development of newer diagnostic tools will have a major public health impact. This commitment may also provide an opportunity for improvement and save lives of many children at-risk. In addition, rehabilitation for those with disabilities needs to be included in conjunction with the capacity building. While challenges remain, a synergistic effort combining all the above-mentioned parameters can be helpful in monitoring and control of JEV dynamics. The ultimate goal is to reduce India’s frighteningly high pediatric mortality rate. Restoring the vaccine deficit by scaling up the currently available interventions would be essential towards India’s roadmap to development.
